# SLC38A9 Regulation Affects Hippocampal Neuronal Autophagy: A Potential Alzheimer's Therapeutic Approach by Suppressing Alzheimer's Disease‐Related Protein Deposition

**DOI:** 10.1002/cns.70823

**Published:** 2026-03-11

**Authors:** Yixin Chen, Xueying Ji, Jiaxiu Zhao, Zhijun Bao, Yiqin Huang

**Affiliations:** ^1^ Department of Geriatrics Huadong Hospital Affiliated to Fudan University Shanghai China; ^2^ Shanghai Key Laboratory of Clinical Geriatric Medicine Shanghai China; ^3^ Shanghai Institute of Geriatrics and Gerontology Shanghai China; ^4^ Department of General Practice Huadong Hospital Affiliated to Fudan University Shanghai China

**Keywords:** alzheimer's disease, autophagy, mTOR/ULK‐1, SLC38A9

## Abstract

**Aims:**

Impaired autophagy‐mediated clearance of Alzheimer's disease (AD)‐related proteins is a critical event in AD pathogenesis. SLC38A9, a member of the Solute Carrier 38 family, acts as an arginine sensor and plays an important role in regulating autophagy. Although the activation of autophagy regulated by the SLC38A9 may have a mitigating effect on AD, this aspect still awaits further exploration.

**Methods:**

APP/PS1 mouse models and HT22 cells treated with amyloid‐β 25–35 (Aβ_25–35_) were transduced with vectors to evaluate the effect of SLC38A9 in AD.

**Results:**

We show that decreasing SLC38A9 could promote the hippocampal neuronal autophagic clearance of AD‐related proteins, reduce neuronal apoptosis, and improve cognitive function.

**Conclusion:**

Our results demonstrate SLC38A9 is involved in AD‐related pathology and its cognitive impairment, and may offer new therapeutic targets to AD.

## Graphical abstract

This study demonstrates that SLC38A9 contributes to cognitive impairment in Alzheimer's disease (AD). By impairing autophagic activity in hippocampal neurons, SLC38A9 inhibits the clearance of pathological proteins, thereby exacerbating synaptic damage and neuronal loss. Collectively, these findings identify SLC38A9 as a potential therapeutic target for mitigating cognitive decline in AD, via SLC38A9‐mediated autophagic clearance of pathological proteins..

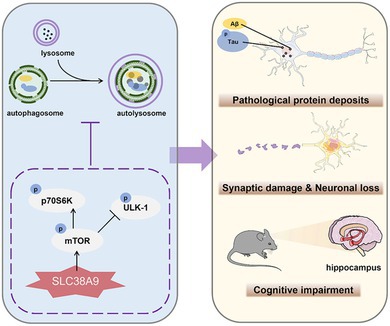

## 1 Introduction

Alzheimer's disease (AD) is a chronic neurodegenerative disorder characterized by cognitive decline and accounts for 60%–80% of dementia cases [1]. The accumulation of β‐amyloid (Aβ) peptides and hyperphosphorylated tau (p‐Tau) proteins results in the formation of senile plaques and neurofibrillary tangles (NFTs). These pathological changes lead to neurotoxicity and neuronal loss [2, 3], ultimately impairing memory and learning.

Impaired autophagy has been strongly linked to neurodegenerative diseases, cancer, and metabolic disorders [4, 5]. When autophagy is disrupted in AD, it fails to efficiently eliminate accumulated Aβ plaques and p‐Tau proteins [6]. Experimental evidence indicates that enhancing mTOR activity using rapamycin facilitates tau clearance via autophagy and improves cognitive performance in mice [7], suggesting that autophagy activation may represent a potential treatment strategy for AD.

Solute Carrier Family 38 Member 9 (SLC38A9) is a lysosomal transmembrane protein activated by L‐arginine [8] and involved in the upregulation of mTOR signaling and the inhibition of autophagy [9, 10, 11, 12]. SLC38A9 expression is significantly upregulated in neurons of AD patients [13], and this increase is associated with AD pathology and the progression of dementia development [14, 15]. However, the role of SLC38A9 in AD‐related cognitive impairment remains unclear. Our study explored the mechanisms underlying the role of SLC38A9 in AD.

## 2 Materials and Methods

### 2.1 Animals Model

Ten‐month‐old male B6C3‐Tg (APPswe/PSEN1dE9)/Nju double transgenic mice (APP/PS1) (genotype: (APPswe) T, (PSEN1dE9) T) and age‐matched wild‐type (WT) male mice were purchased from Cyagen Biosciences (Suzhou, China).

### 2.2 Adeno‐Associated Virus (AAV) Vector Packaging In Vivo

For SLC38A9 knockdown, a specific short hairpin RNA (shRNA) targeting mouse SLC38A9 and a non‐targeting control sequence were inserted into the AAV‐BBB2.0 vector (U6 promoter; Hanbio Biotechnology, Shanghai, China). The constructs were validated by sequencing. The final viral titers were 1.0 × 10^12^ genome copies/mL for both SLC38A9 shRNA (shSLC38A9) and the non‐targeting control (shNC). AAV‐BBB2.0 vectors were administered via tail vein injection (100 μL per mouse), utilizing the vector's ability to cross the blood–brain barrier [16]. The tail vein injection of AAV‐BBB2.0 did not alter SLC38A9 expression in peripheral organs (Figure S10A–C). Furthermore, this intervention has no significant impact on liver and kidney function of the mice (Figure S11A–F).

### 2.3 Animal Treatment

To evaluate the effect of SLC38A9 decrease, mice received injections of shSLC38A9. Following behavioral testing, mice were euthanized. Brains were harvested and divided into two parts. One portion was used to isolate hippocampal tissue and stored at −80°C for further analysis. The other portion was fixed in 4% paraformaldehyde (PFA) for 48 h and embedded in paraffin.

### 2.4 Mice Cognitive Function Test

#### 2.4.1 Morris Water Maze (MWM) Test

The test consisted of two phases based on previous studies [17]. During the 5‐day hidden platform phase, escape latency was recorded. In the probe trial, the latency to first arrival, time spent in the target quadrant, and number of platform crossings were measured.

#### 2.4.2 Novel Object Recognition (NOR) Test

This test was also divided into two phases [18]. In the first phase, two identical objects were placed in the testing box. Each mouse was allowed to explore for 10 min, followed by a 1‐h rest period. In the second phase, one object was replaced with a novel object, and mice were allowed to explore for 5 min. Behavior was recorded, and the novel object preference index was calculated as the time exploring the novel object divided by the total time spent exploring both objects.

### 2.5 Transcriptome Sequencing Analysis

RNA sequencing was performed by Novogene (Beijing, China). Total RNA was extracted from hippocampal tissue to construct cDNA libraries and conduct RNA sequencing. Gene expression levels were quantified, and differentially expressed genes (DEGs) were identified using R software, with criteria of fold change ≥ 1.0 and *p*‐value ≤ 0.05. Gene Ontology (GO) and Kyoto Encyclopedia of Genes and Genomes (KEGG) pathway analyses were conducted using the clusterProfiler package in R.

### 2.6 H&E and Nissl Staining

Brains fixed in 4% formalin were processed, dehydrated, embedded in paraffin, and sectioned coronally. Sections were stained with H&E or 0.5% tolyl violet. Slides were then dehydrated in graded ethanol, cleared in xylene, and mounted with neutral resin. Morphological damage and neuronal injury were examined under an optical microscope (IX53, Olympus, Tokyo, Japan).

### 2.7 Lentivirus Plasmid Construction

shRNA sequences targeting SLC38A9 and a control sequence were designed by Hanbio Biotechnology (Shanghai, China). Lentiviral vectors encoding shSLC38A9 (with GFP) and a non‐targeting control shRNA (shNC) were constructed. The gene knockdown efficiency data were provided in Figure S3A–C.

### 2.8 Cell Culture and Treatment

Mouse hippocampal neuron HT22 cells (Chinese Academy of Sciences, Shanghai, China) were cultured and transfected with lentivirus according to the manufacturer's instructions. SLC38A9 knockdown was performed, followed by treatment with 100 μM L‐arginine (A600205, Sangon Biotech, China) [19] or 3 mM 3‐methyladenine (3‐MA) (M9281, Sigma‐Aldrich, USA) for 4 h. Cells were then co‐incubated with 40 μM Aβ_25–35_ for 48 h to simulate the in vitro AD pathological environment based on prior work [20].

### 2.9 Terminal Deoxynucleotidyl Transferase dUTP Nick End Labeling (TUNEL)

The TUNEL assay was conducted following a previously established protocol [17]. Images were acquired using a confocal microscope (Leica SPE), and ImageJ software (NIH, USA) was used for analysis.

### 2.10 mRFP‐GFP‐LC3 Fluorescence Microscopy

HT22 cells were infected with tandem fluorescent‐tagged mRFP‐GFP‐LC3 adenovirus (Hanbio Biotechnology, Shanghai, China) to monitor autophagosome and autolysosome formation and assess autophagic flux. After 24 h of infection, cells were treated accordingly, fixed with 4% PFA, and stained with DAPI to visualize nuclei. Images were captured using a confocal microscope (Leica SPE) and analyzed with ImageJ (NIH, USA).

### 2.11 Immunohistochemistry (IHC) and Immunofluorescence (IF) Staining

Hippocampal tissues were dissected from mice, fixed in 4% PFA, and embedded in paraffin. The tissues were then sectioned into slices. For immunohistochemistry (IHC), hippocampal sections were incubated with anti‐Aβ and anti‐p‐Tau antibodies. For immunofluorescence (IF), HT22 cells were incubated with primary antibodies against SLC38A9 and LC3, derived from different species. Images were observed under a microscope and captured using a confocal microscope (Leica SPE). Image analysis was conducted using ImageJ software (NIH, USA).

### 2.12 Western Blotting

Immunoblotting was performed according to our previously established protocol [17]. The information of antibodies is in Table S1.

### 2.13 Statistical Analysis

Statistical analyses were performed using GraphPad Prism 10 software. Data from at least three independent experiments were analyzed using Student's *t*‐test (for comparisons between two groups) or one‐way analysis of variance (ANOVA, for multiple groups). Results are presented as mean ± standard error of the mean (SEM). A *p*‐value less than 0.05 was considered statistically significant.

## 3 Results

### 3.1 Increased Expression of SLC38A9 in Hippocampus of AD

**FIGURE 1:**
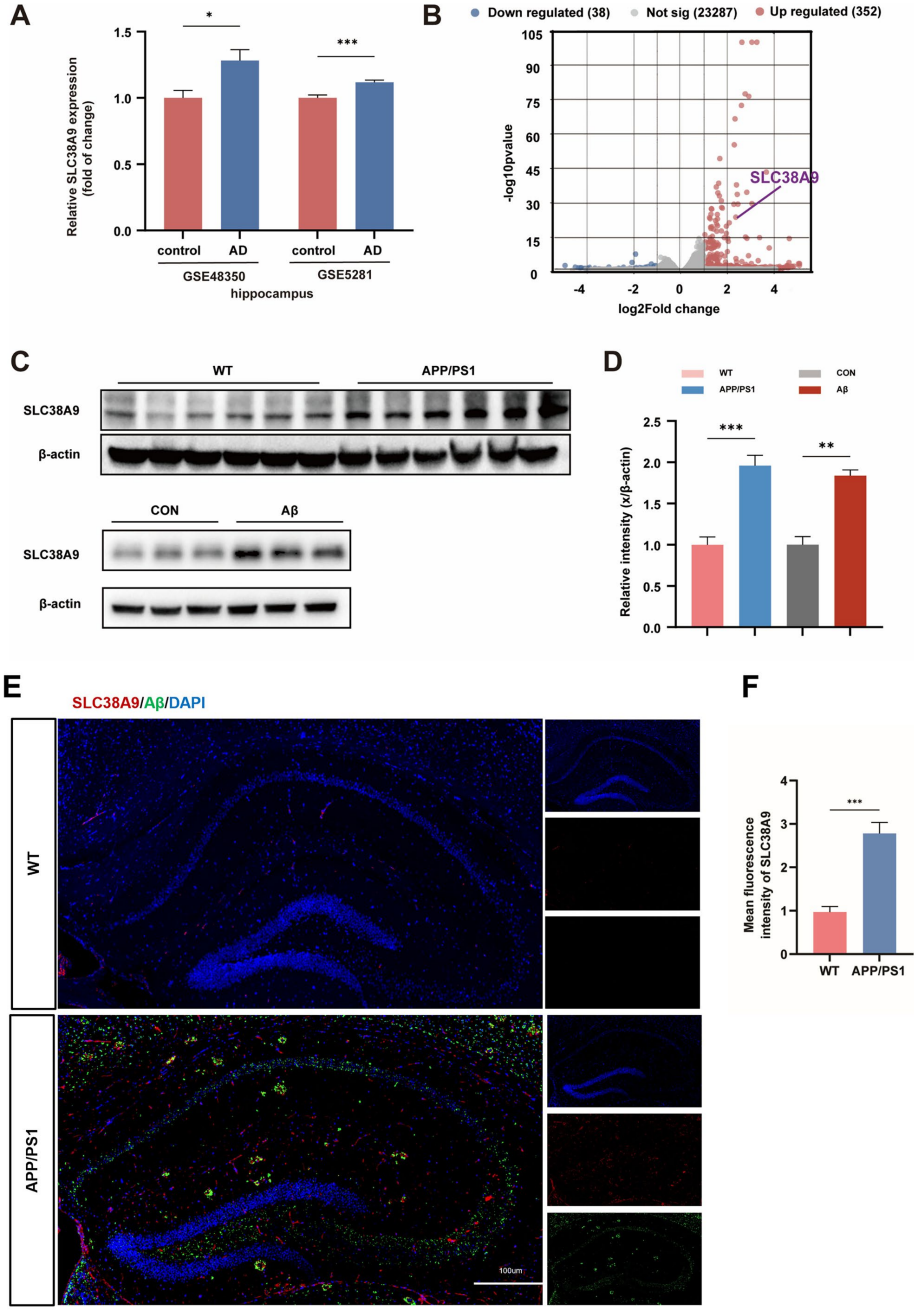
Increased expression of SLC38A9 in hippocampal neuron of Alzheimer's disease (AD). (A) Transcriptional up‐regulation of SLC38A9 in the hippocampus of the GSE48350 database (control, *n* = 16; AD patients, *n* = 19) and the GSE5281 database (control, *n* = 13; AD patients, *n* = 10). (B) Total numbers of genes in the hippocampus tissues that were upregulated or downregulated and the volcano plot of DEGs between the APP/PS1 mice and sham group. (*n* = 6). (C, D) Representative immunoblotting bands and quantification of SLC38A9/β‐Actin in the mice hippocampus (*n* = 6) and HT22 cells (*n* = 3). (E, F) Representative merged immunofluorescence images of Aβ, SLC38A9 and DAPI in the hippocampal region. Scale bar = 100 μm. Quantification of the SLC38A9 fluorescence intensity using ImageJ software. (*n* = 6). Data are presented as the mean ± SEM. *p < 0.05, **p < 0.01, ***p < 0.001 vs. sham group.

We first analyzed human transcriptomic datasets to assess changes in SLC38A9 expression in AD. Analysis of the GSE48350 and GSE5281 datasets showed significantly elevated SLC38A9 mRNA levels in the hippocampus of AD patients compared to controls (Figure 1A). RNA‐seq analysis of 10‐month‐old APP/PS1 hippocampus revealed 352 upregulated and 38 downregulated genes, with SLC38A9 among the significantly increased ones (Figure 1B). Immunoblotting results showed that SLC38A9 protein levels were significantly increased in both APP/PS1 hippocampus and Aβ_25–35_‐treated HT22 cells (Figure 1C,D). Figure S5A–H presents comparative data on the modeling performance of Aβ_25–35_ versus Aβ_1–42_, supporting our choice of Aβ_25–35_. Moreover, IF results showed increased fluorescence intensity of SLC38A9 in APP/PS1 hippocampal sections (Figure 1E,F). Aβ levels in the brain tissue of WT and APP/PS1 mice measured by IHC and IF (Figure S1A–D). These findings suggest a potential association between AD pathological features and SLC38A9 regulation.

### 3.2 SLC38A9 Is Involved in the Cognitive Impairment in APP/PS1 Mice

**FIGURE 2:**
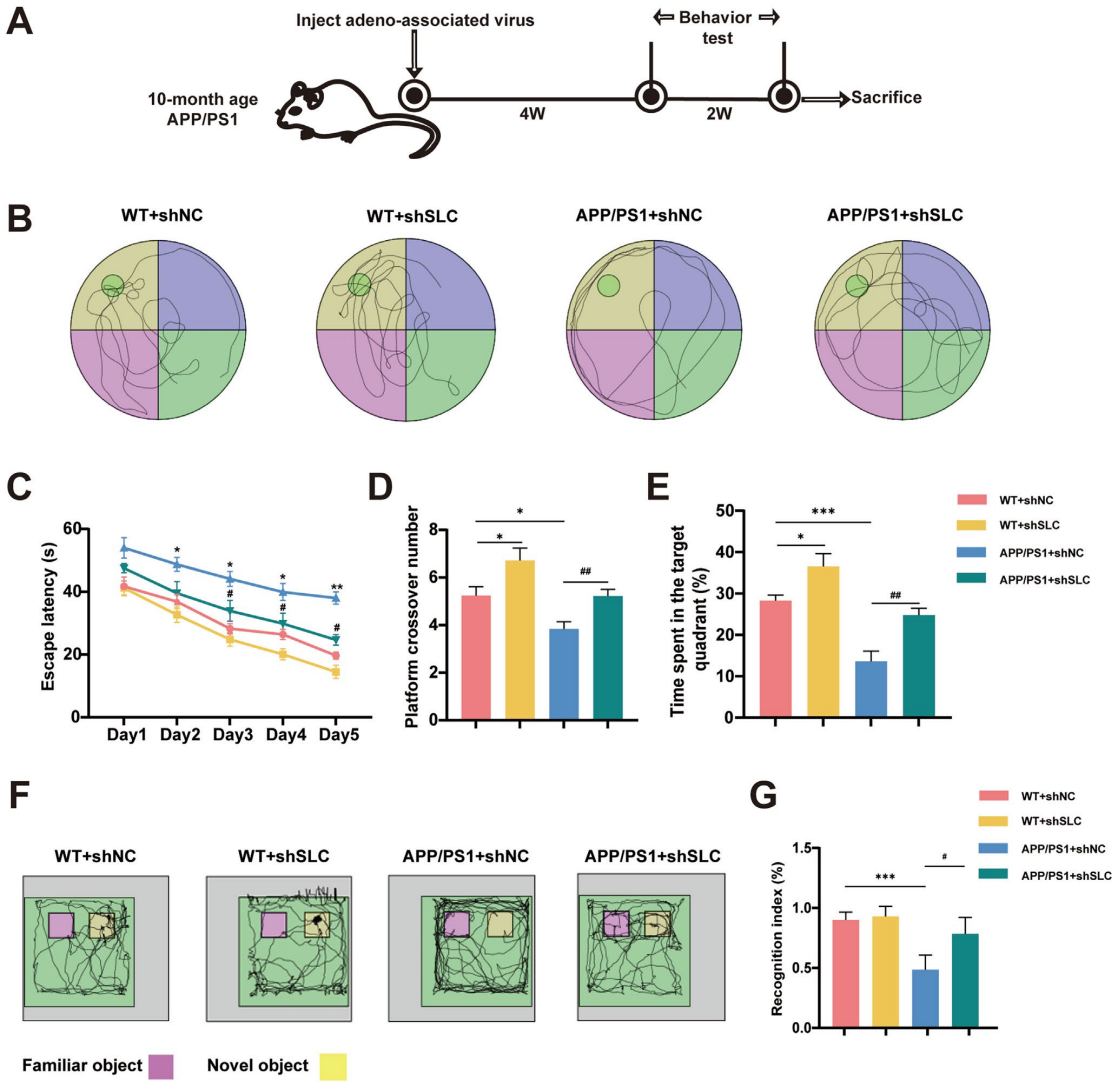
SLC38A9 is involved in the cognitive impairment in APP/PS1 mice. (A) Schematic diagram of the experimental procedure. (B) Representative exploring traces of mice in different groups during the probe trial. (C) The escape latency of mice to reach the platform during the acquisition test. (D) The numbers of platform crossings in the probe trial. (E) Percentage of time spent in the target quadrant of mice in the probe trial. (F) Representative trajectories in the NOR (Novel object recognition) test. (G) Recognition index of the novel object in the NOR. *n* = 8–10 per group. Data are presented as the mean ± SEM. *p < 0.05, ***p < 0.001 vs. sham group; ^#^ *p* < 0.05, ^##^ *p* < 0.01 vs. the APP/PS1 + shNC mice group.

To assess the role of SLC38A9 in cognitive function, we reduced its expression in APP/PS1 mice via tail vein injection of virus. After 4 weeks, behavioral tests including the Morris Water Maze (MWM) and Novel Object Recognition (NOR) were conducted (Figure 2A). SLC38A9 inhibition significantly reduced escape latency and increased both the time spent in the target quadrant and the frequency of platform crossings in the MWM (Figure 2B–E). There was no significant difference in swimming speed among groups, indicating no motor or visual deficits. In the NOR test, the AD group showed reduced preference for the novel object, while decreased SLC38A9 increased exploratory time for the novel object (Figure 2F,G). These results indicate that SLC38A9 affects cognitive decline in AD.

### 3.3 SLC38A9 Promotes to Neuronal Damage and AD‐Related Proteins Deposition in Hippocampal Neuron of AD

**FIGURE 3:**
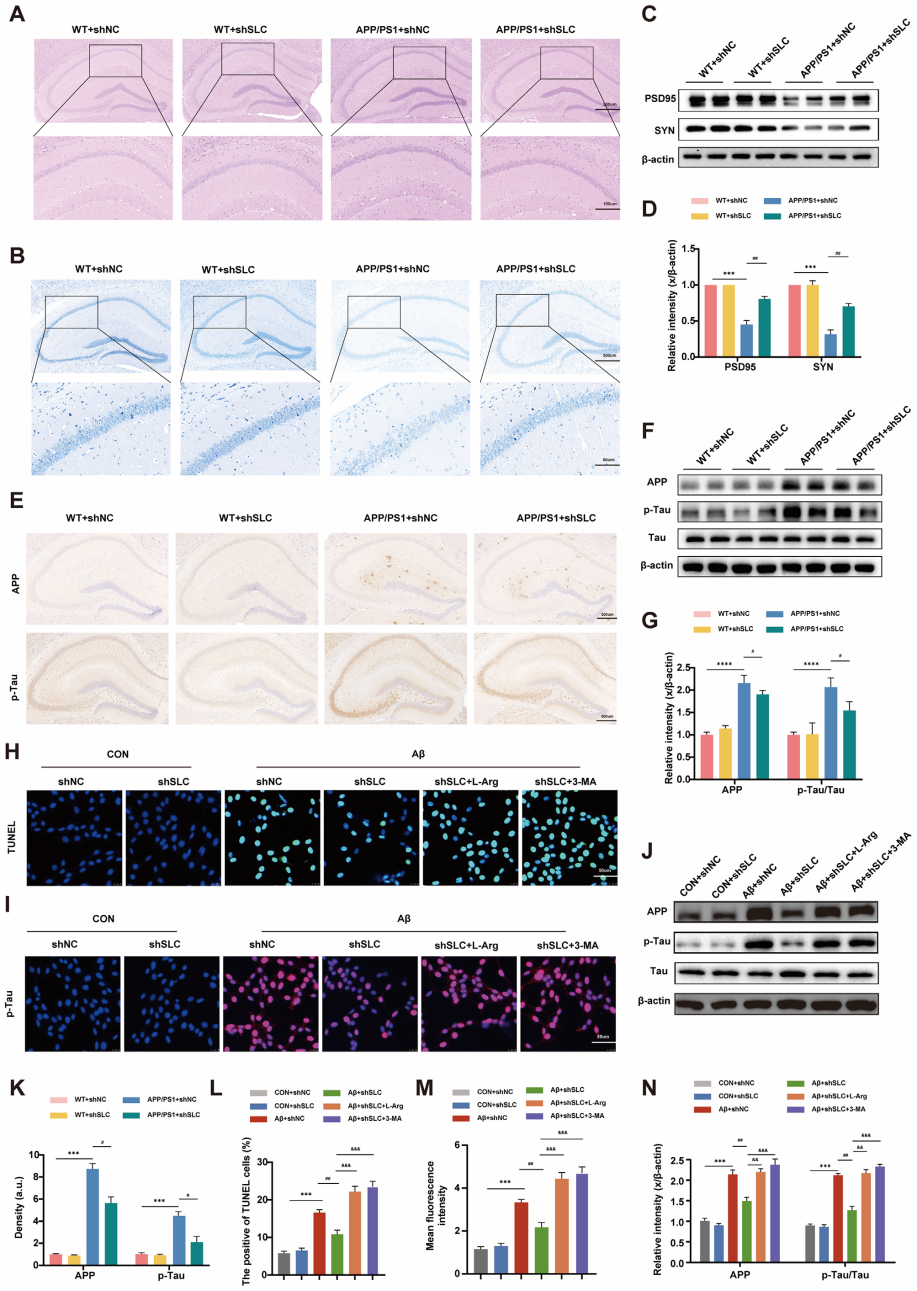
SLC38A9 Contributes to neuronal damage and AD‐related proteins deposition in hippocampal neuron of AD. (A) Representative H&E staining of the mice hippocampus. Scale bar = 200 μm (upper panel) or 100 μm (lower panel). (*n* = 6). (B) Representative Nissl staining in the mice hippocampus. Scale bar = 200 μm (upper panel) or 50 μm (lower panel) (*n* = 6). (C) Representative immunoblotting bands of PSD95 and SYN in the hippocampus of mice. (D) Quantification of PSD95/β‐actin and SYN/β‐actin in the (C). (*n* = 6). (E) IHC staining of APP and p‐Tau in mice hippocampus. Scale bar = 300 μm. (F) Representative immunoblotting bands of APP/β‐actin and p‐Tau/Tau in the mice hippocampus. (G) Quantification of APP/β‐Actin and p‐Tau/Tau in the (F) (*n* = 6). (H) Representative merged immunofluorescence images of apoptosis cells (Green) and DAPI in HT22 cells. Scale bars = 50 μm. (I) Representative merged immunofluorescence images of p‐Tau (Red) and DAPI in HT22 cells. Scale bar = 50 μm. (J) Representative immunoblotting bands of APP/β‐Actin and p‐Tau/Tau in HT22 cells. (K) Quantification of the APP and p‐Tau intensity area in E using ImageJ software. (*n* = 6). (L) Quantification the proportion of TUNEL positive cells in H using ImageJ software. (*n* = 3). (M) Quantification of the fluorescence intensity in I using ImageJ software. (*n* = 3). (N) Quantification of APP/β‐Actin and p‐Tau/Tau in the J. (*n* = 3). Data are presented as the mean ± SEM. **p < 0.01, ***p < 0.001, ****p < 0.0001 vs. sham group; ^#^ *p* < 0.05, ^##^ *p* < 0.01 vs. the APP/PS1 + shNC mice group or the Aβ + shNC cells group; ^&&^ *p* < 0.01, ^&&&^ *p* < 0.001 vs. the Aβ + shSLC cells group.

H&E staining of hippocampal sections revealed nuclear condensation and increased staining in APP/PS1 mice, which were alleviated by SLC38A9 decrease (Figure 3A). Nissl staining showed reduced Nissl‐positive neurons and disorganized arrangement in APP/PS1 mice, indicating neuronal damage, which was partially restored by SLC38A9 reduced (Figure 3B). Immunoblotting analysis showed reduced postsynaptic density 95 (PSD95) and synapsin protein (SYN) expression in APP/PS1 mice, which was significantly reversed by SLC38A9 reduced (Figure 3C,D). To further evaluate neuronal protection, we examined apoptosis in Aβ_25–35_‐treated HT22 cells using TUNEL staining. Aβ_25–35_ significantly increased apoptosis, while SLC38A9 knockdown reduced the proportion of apoptotic cells. This effect was reversed by L‐arginine treatment (Figure 3H,L). In primary mouse hippocampal neurons, these treatments had the same effect (Figure S7A–C). We also conduct MTS Cell Proliferation Assay, the MTS results corroborate the TUNEL findings, showing concordant changes in viability across the experimental groups (Figure S2).

IHC staining confirmed that APP and p‐Tau/Tau levels were significantly increased in APP/PS1 mice hippocampal sections, while SLC38A9 reduction reversed these changes (Figure 3E,K). Immunoblotting analysis shows the reduced expression of APP and p‐Tau in hippocampal area following decrease SLC38A9 (Figure 3F,G). Similarly, in Aβ_25–35_‐treated HT22 cells, inhibition of SLC38A9 decreased the levels of AD‐related proteins, while L‐arginine reversed this effect (Figure 3J,N). IF staining also showed a significant reduction in p‐Tau protein expression following SLC38A9 decrease (Figure 3I,M). Similarly, SLC38A9 reduction treatment had the same effect in Aβ_1–42_‐treated HT22 cell (Figure S8A–F). These in vivo and in vitro results support the role of SLC38A9 in hippocampal neuronal damage and AD‐related proteins deposition.

### 3.4 SLC38A9 Is Involved in Autophagy Inhibition in Hippocampal Neuron of AD

**FIGURE 4:**
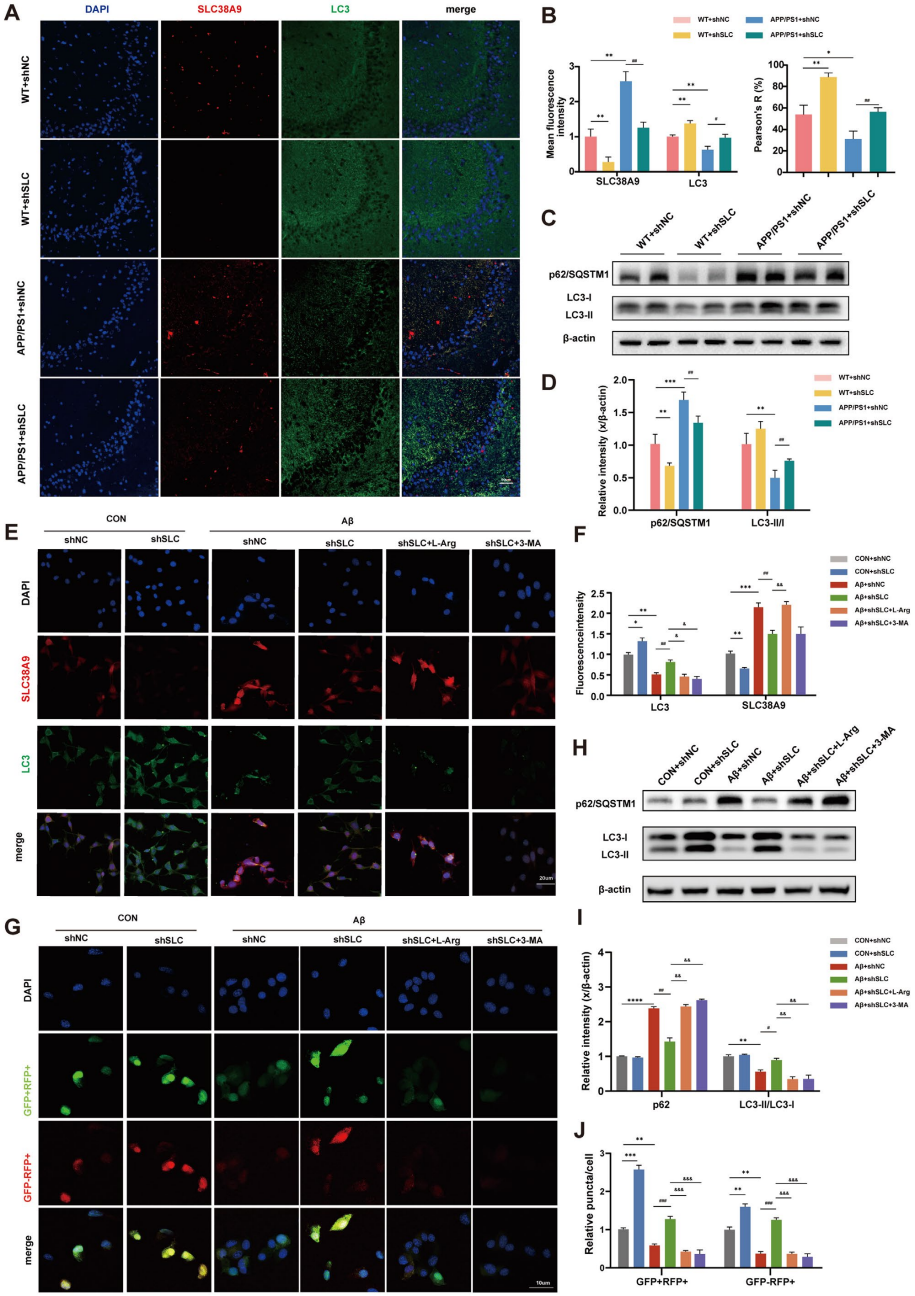
SLC38A9 Is involved in autophagy inhibition in hippocampal neuron of AD. (A, B) Representative merged immunofluorescence images of SLC38A9, LC3 and DAPI in the hippocampal region. Scale bar = 50 μm. Quantification of the SLC38A9 and LC3 fluorescence intensity using ImageJ software and calculate the Pearson's (%). (*n* = 6). (C, D) Representative immunoblotting bands and quantification of p62/SQSTM1/β‐Actin and LC3‐II/I in mice hippocampus. (*n* = 6). (E) Representative merged immunofluorescence images of SLC38A9, LC3 and DAPI in HT22 cells. Scale bar = 20 μm. (F) Quantification of the SLC38A9 and LC3 fluorescence intensity using ImageJ software. (*n* = 3). (G) Representative confocal images of LC3 puncta in HT22 cells expressing mRFP‐GFP‐LC3. Scale bar = 10 μm. (H, I) Representative immunoblotting bands and quantification of p62/SQSTM1/β‐Actin and LC3‐II/I in HT22 cells. (*n* = 3). (J) Quantification of the relative LC3 puncta in (G) using ImageJ software. (*n* = 3). Data are presented as the mean ± SEM. *p < 0.05, **p < 0.01, ***p < 0.001, ****p < 0.0001 vs. sham group; ^#^ *p* < 0.05, ^##^ *p* < 0.01, ^###^ *p* < 0.001 vs. the APP/PS1 + shNC mice group or the Aβ + shNC cells group; ^&^ *p* < 0.05, ^&&^ *p* < 0.01, ^&&&^ *p* < 0.001 vs. the Aβ + shSLC cells group.

Autophagy was markedly impaired in the hippocampus of APP/PS1 mice, as indicated by elevated p62/SQSTM1 levels and decreased LC3‐II/I ratio. IF analysis showed increased LC3 fluorescence intensity in the hippocampus following SLC38A9 knockdown (Figure 4A,B), suggesting enhanced autophagic activity. Immunoblotting analysis also shows that SLC38A9 reduction significantly reversed these changes, increasing the LC3‐II/I ratio and reducing p62/SQSTM1 levels (Figure 4C,D).

In vitro experiments further confirmed that Aβ_25–35_ impaired autophagy function. Under Aβ_25–35_ treatment, p62/SQSTM1 levels increased, while the LC3‐II/I ratio decreased. However, SLC38A9 decrease reversed these effects. IF staining showed a significant increase in LC3 protein expression following SLC38A9 decrease (Figure 4E,F). Immunoblotting analysis also shows that SLC38A9 reduction significantly reversed these changes, increasing the LC3‐II/I ratio and reducing p62/SQSTM1 levels; this effect of decreased SLC38A9 was altered by the addition of L‐arginine or 3‐methyladenine (3‐MA) (autophagy inhibitor) (Figure 4H,I). The differences in autophagy dynamics between in vivo and in vitro are provided in the Figure S4A–D.

To evaluate autophagy activation after SLC38A9 knockdown, we used mRFP‐GFP tandem fluorescent LC3 to track the generation of autophagosomes and autolysosomes and to quantify autophagic flux. The acidic milieu of lysosomes quenches GFP, so autophagosomes show both GFP and RFP signals, while autolysosomes retain only RFP. Initially, we observed that the numbers of autophagosomes and autolysosomes were reduced in Aβ_25–35_‐treated cells, indicating inhibited autophagic flux. However, SLC38A9 knockdown significantly increased the numbers of autophagosomes and autolysosomes. We performed staining and quantitative analysis using LysoTracker between the SLC38A9 knockdown group and the control group, indicating that lysosomal pH was unaffected and that SLC38A9 knockdown does not impair lysosomal acidification; this finding suggests that the observed changes in autophagic flux are not caused by lysosomal deacidification (Figure S12A,B). We first observed that, relative to the control group, Aβ_25–35_ exposure reduced both autophagosome and autolysosome numbers, implying that autophagic flux is blocked at an early step. In contrast, SLC38A9 knockdown markedly increased the numbers of autophagosomes and autolysosomes in HT22 cells under Aβ_25–35_, compared with the empty‐vector control. This effect was abolished following treatment with L‐arginine or 3‐MA (Figure 4G,J). Similarly, knockdown of SLC38A9 exerted analogous effects in Aβ_1–42_‐treated HT22 cells (Figure S9A–F), and comparable outcomes were observed in primary mouse hippocampal neurons subjected to the same intervention (Figure S7B–D). Furthermore, our supplementary data on bafilomycin A1 (Baf‐A1) and chloroquine (CQ) intervention (Figure S6A–D) are consistent with the above findings, thus validating our conclusions. These in vivo and in vitro results demonstrate that SLC38A9 inhibits hippocampal neuron autophagy function.

### 3.5 SLC38A9 Promotes to AD‐Related Proteins Deposition in Hippocampal Neurons Through Inhibition of Neuronal Autophagy

To verify that the effect of SLC38A9 to intervene abnormal protein deposition leading to neuronal damage via autophagy inhibition, we detected the AD‐related proteins levels and cell apoptosis proportion in vitro experiments. As expected, co‐treatment with 3‐MA reversed the effect of autophagy function increase by SLC38A9 inhibition and increased the levels of AD‐related proteins (Figures 3J,N and 4H,I). IF staining also showed a significant increase in cell apoptosis (Figure 3H,L) and p‐Tau protein expression (Figure 3I,M) after 3‐MA treatment. These results indicate that SLC38A9 inhibits hippocampal neuronal autophagy to AD‐related proteins deposition leading to neuronal damage.

### 3.6 SLC38A9 Is Involved in Cognitive Impairment of AD by Activating the mTOR Signaling Pathway

**FIGURE 5:**
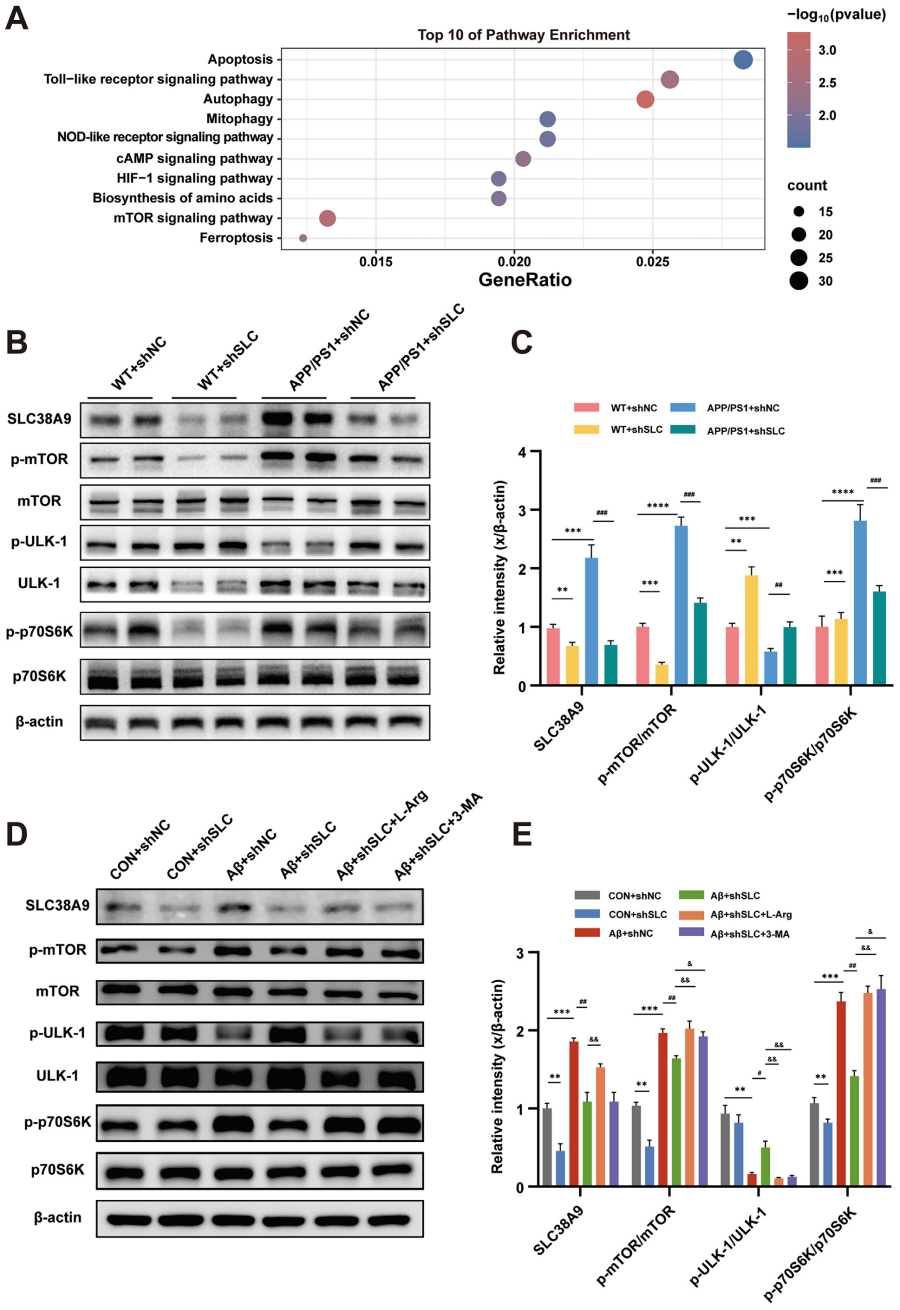
SLC38A9 is involved in the mTOR signaling pathway in hippocampal neuron of AD. (A) The intersection of DEGs for the hippocampus in APP/PS1 + shSLC38A9 mice vs. APP/PS1+ shNC mice was analyzed by KEGG enrichment and the top 10 enriched pathways are shown. (*n* = 6). (B, C) Representative immunoblotting bands and quantification of SLC38A9, p‐mTOR/mTOR, p‐ULK‐1/ULK‐1 and p‐p70S6K/p70S6K in mice hippocampus. (*n* = 6). (D, E) Representative immunoblotting bands and quantification of SLC38A9, p‐mTOR/mTOR, p‐ULK‐1/ULK‐1 and p‐p70S6K/p70S6K in HT22 cells. (*n* = 3). Data are presented as the mean ± SEM. **p < 0.01, ***p < 0.001, ****p < 0.0001 vs. sham group; ^#^ *p* < 0.05, ^##^ *p* < 0.01, ^###^ *p* < 0.001 vs. the APP/PS1 + shNC mice group or the Aβ + shNC cells group; ^&^ *p* < 0.05, ^&&^ *p* < 0.01 vs. the APP/PS1 + shSLC mice group or the Aβ + shSLC cells group.

To explore the mechanisms by which SLC38A9 regulates autophagy, RNA‐seq analysis was performed. KEGG pathway enrichment of differentially expressed genes (DEGs) for the hippocampus in APP/PS1 + shSLC38A9 mice vs. APP/PS1 + shNC mice revealed that the mTOR signaling pathway was most significantly affected by SLC38A9 (Figure 5A). Immunoblotting confirmed effective SLC38A9 knockdown in the hippocampus of transfected mice. In APP/PS1 mice, SLC38A9, p‐mTOR/mTOR, and p‐p70S6K/p70S6K levels were elevated, while p‐ULK‐1/ULK‐1 expression was reduced. These alterations were reversed following SLC38A9 inhibition (Figure 5B,C).

We further validated these findings in vitro. In Aβ_25–35_‐treated HT22 cells, SLC38A9 reduction significantly reduced the levels of p‐mTOR/mTOR and p‐p70S6K/p70S6K, while p‐ULK‐1/ULK‐1 expression was increased (Figure 5D,E). L‐arginine treatment inhibited the reduction of SLC38A9, the reduction of p‐mTOR/mTOR and p‐p70S6K/p70S6K, and blocked the increase of p‐ULK‐1/ULK‐1. 3‐MA treatment increased p‐mTOR/mTOR and p‐p70S6K/p70S6K expression and decreased p‐ULK‐1/ULK‐1 levels without significantly altering SLC38A9 expression. These findings confirm that SLC38A9 modulated the mTOR signaling pathway in AD.

## 4 Discussion

SLC38A9 is part of the amino acid‐sensing system and could be activated by L‐arginine. Evidence increasingly supports its role as a therapeutic target in various neurological disorders [21, 22, 23]. Through bioinformatics analysis, we found that SLC38A9 expression is significantly altered in the hippocampal regions of AD patients. We also observed upregulation of SLC38A9 in both in vivo and in vitro AD models. These findings led us to hypothesize that SLC38A9 may contribute to hippocampal neuronal pathology.

We observed that in APP/PS1 mice, SLC38A9 knockdown reduced cognitive impairments. Neuronal damage is closely associated with cognitive decline in AD [24, 25]. Loss of hippocampal synapses, a hallmark contributing to cognitive deficits in AD models and patients, involves critical proteins such as the postsynaptic PSD95 and presynaptic Synaptophysin. We found that SLC38A9 knockdown robustly enhanced the protein abundance in APP/PS1 mice, thereby improving learning and memory. Excess Aβ accumulation contributes to synaptic dysfunction and neuronal loss, resulting in memory deficits [26, 27]. Elevated p‐Tau leads to neurodegeneration when pathological thresholds are exceeded. Pathological protein accumulation is toxic to hippocampal neurons, leading to hippocampal neuronal loss and cognitive deficits. Our data confirmed SLC38A9 knockdown significantly reduced this accumulation. We considered that SLC38A9 is significantly correlated with the pathological accumulation of aberrant amyloid proteins and hippocampal neuronal damage.

Recent studies show that autophagy impairment in AD contributes to reduced clearance of Aβ and p‐Tau [28, 29, 30]. LC3‐II/I ratio is positively correlated with autophagic activity, while p62/SQSTM1 is inversely correlated [31]. Our results revealed that autophagy was significantly inhibited in APP/PS1 hippocampus and Aβ_25–35_‐treated HT22 cells. As previously reported, SLC38A9 decrease restores autophagy function [32]. L‐arginine was administered to activate any remaining SLC38A9 and thereby offset the effects of the knockdown, given its arginine sensitivity. The compound 3‐MA functions as an autophagy suppressor, reducing autophagosome formation and autophagic flux. After treatment with L‐arginine or 3‐MA, the effects of restoring autophagy function and attenuating AD‐related pathology are reversed. These findings further support that SLC38A9 contributes to cognitive impairment in AD via suppressing hippocampal neuronal autophagic clearance of pathological protein.

AD is not merely characterized by abnormal protein accumulation, but also by a lysosomal signaling disorder that is inextricably intertwined with the aberrant activation of mTORC1, and this lysosomal signaling disorder is closely related to autophagic dysfunction. Studies have shown that SLC38A9 can activate mTORC1, thereby indirectly modulating autophagy [11]. KEGG pathway analysis revealed that most DEGs were enriched in the mTOR signaling pathway. mTOR signaling acts as a key modulator in protecting against AD‐induced cognitive impairment [33, 34]. Activated mTOR regulates autophagy through downstream effectors, including Unc‐51‐like kinase 1 (ULK‐1) and p70S6 kinase (p70S6K) [35, 36], involved in autophagy regulation and abnormal protein clearance in AD [37, 38]. We found that SLC38A9 expression inhibition reduced the phosphorylation of mTOR and p70S6K, and increased the phosphorylation of ULK‐1. Furthermore, the effects of SLC38A9 decrease were abolished by co‐treatment with L‐arginine or 3‐MA, indicating that inhibition of autophagy by SLC38A9 is dependent on the mTOR pathway. Based on these results, we propose that the mTOR pathway is essential for the inhibition in autophagy effects of SLC38A9 in AD models.

In this study, we demonstrated for the first time that SLC38A9 contributes to cognitive impairment in AD by suppressing hippocampal neuronal autophagic clearance of pathological proteins, thereby supporting SLC38A9 as a potential therapeutic target for AD, and since targeted modulation of SLC38A9 enables precise regulation of lysosomal function and mTORC1 activity and autophagy activity, establishing SLC38A9 as a druggable target is a feasible and promising direction for translational research.

## Author Contributions

**Yixin Chen:** writing original draft; **Xueying Ji:** review and editing; **Jiaxiu Zhao:** supervision; **Yiqin Huang and Zhijun Bao:** conceptualization and funding acquisition.

## Funding

This work was supported by the National Natural Science Foundation of China (grant number 82071581), the Shanghai Leading Talent Program (grant number 2022002), the Shanghai Municipal Health Commission Key Support Discipline Program (grant number 2023ZDFC0402), and the China Youth Medical Innovation Research Project (Phase VIII, No. 11).

## Ethics Statement

All animal experiments were approved by the Fudan University School of Life Sciences Research Ethics Committee (permit No. 2022110016S). All procedures were carried out in strict accordance with ARRIVE guidelines.

## Conflicts of Interest

The authors declare no conflicts of interest.

## Supporting information


**Figure S1:** Immunohistochemical (IHC) and immunofluorescence (IF) analysis of Aβ deposition in the hippocampal region of mice from each group.
**Figure S2:** The viability of HT22 cells was determined by MTS assay.
**Figure S3:** Detection of SLC38A9 expression levels in knockdown cells.
**Figure S4:** Temporal changes in autophagy markers following Aβ treatment.
**Figure S5:** Evaluation of the cytotoxic effects of Aβ_1–42_ and Aβ_25–35_ and their impact on Alzheimer's disease‐related protein deposition in HT22 cells.
**Figure S6:** Validation of autophagic flux changes using bafilomycin A1 and chloroquine in the Aβ‐treated cell model.
**Figure S7:** Validation of autophagy flux and apoptosis assays in mouse primary hippocampal neurons.
**Figure S8:** Effects of SLC38A9 knockdown on AD‐related protein expression and cell apoptosis in Aβ_1–42_‐treated HT22 cells.
**Figure S9:** Effects of SLC38A9 knockdown on autophagic activity in Aβ_1‐42_‐treated HT22 cells.
**Figure S10:** SLC38A9 expression in mice liver and kidneys after AAV‐BBB2.0 injection.
**Figure S11:** Serum biochemical indices of wild‐type mice treated with shNC and shSLC.
**Figure S12:** Assessment of lysosomal pH using LysoTracker staining.
**Table S1:** Antibodies.

## Data Availability

The datasets used and/or analyzed during the current study are available from the corresponding author upon reasonable request.
