# Supplementary material for: SLC38A9 Regulation Affects Hippocampal Neuronal Autophagy: A Potential Alzheimer's Therapeutic Approach by Suppressing Alzheimer's Disease‐Related Protein Deposition
Source: CNS Neurosci Ther. 2026 Mar 11;32(3):e70823. doi: 10.1002/cns.70823 (PMC12977986; doi:10.1002/cns.70823)
Supplement: Supplementary file 1 — Figure S1: Immunohistochemical (IHC) and immunofluorescence (IF) analysis of Aβ deposition in the hippocampal region of mice from each group. Figure S2: The viability of HT22 cells was determined by MTS assay. Figure S3: Detection of SLC38A9 expression levels in knockdown cells. Figure S4: Temporal changes in autophagy markers following Aβ treatment. Figure S5: Evaluation of the cytotoxic effects of Aβ1–42 and Aβ25–35 and their impact on Alzheimer's disease‐related protein deposition in HT22 cells. Figure S6: Validation of autophagic flux changes using bafilomycin A1 and chloroquine in the Aβ‐treated cell model. Figure S7: Validation of autophagy flux and apoptosis assays in mouse primary hippocampal neurons. Figure S8: Effects of SLC38A9 knockdown on AD‐related protein expression and cell apoptosis in Aβ1–42‐treated HT22 cells. Figure S9: Effects of SLC38A9 knockdown on autophagic activity in Aβ1‐42‐treated HT22 cells. Figure S10: SLC38A9 expression in mice liver and kidneys after AAV‐BBB2.0 injection. Figure S11: Serum biochemical indices of wild‐type mice treated with shNC and shSLC. Figure S12: Assessment of lysosomal pH using LysoTracker staining. Table S1: Antibodies. [file CNS-32-e70823-s001.doc]

## **Supplementary Figure1**


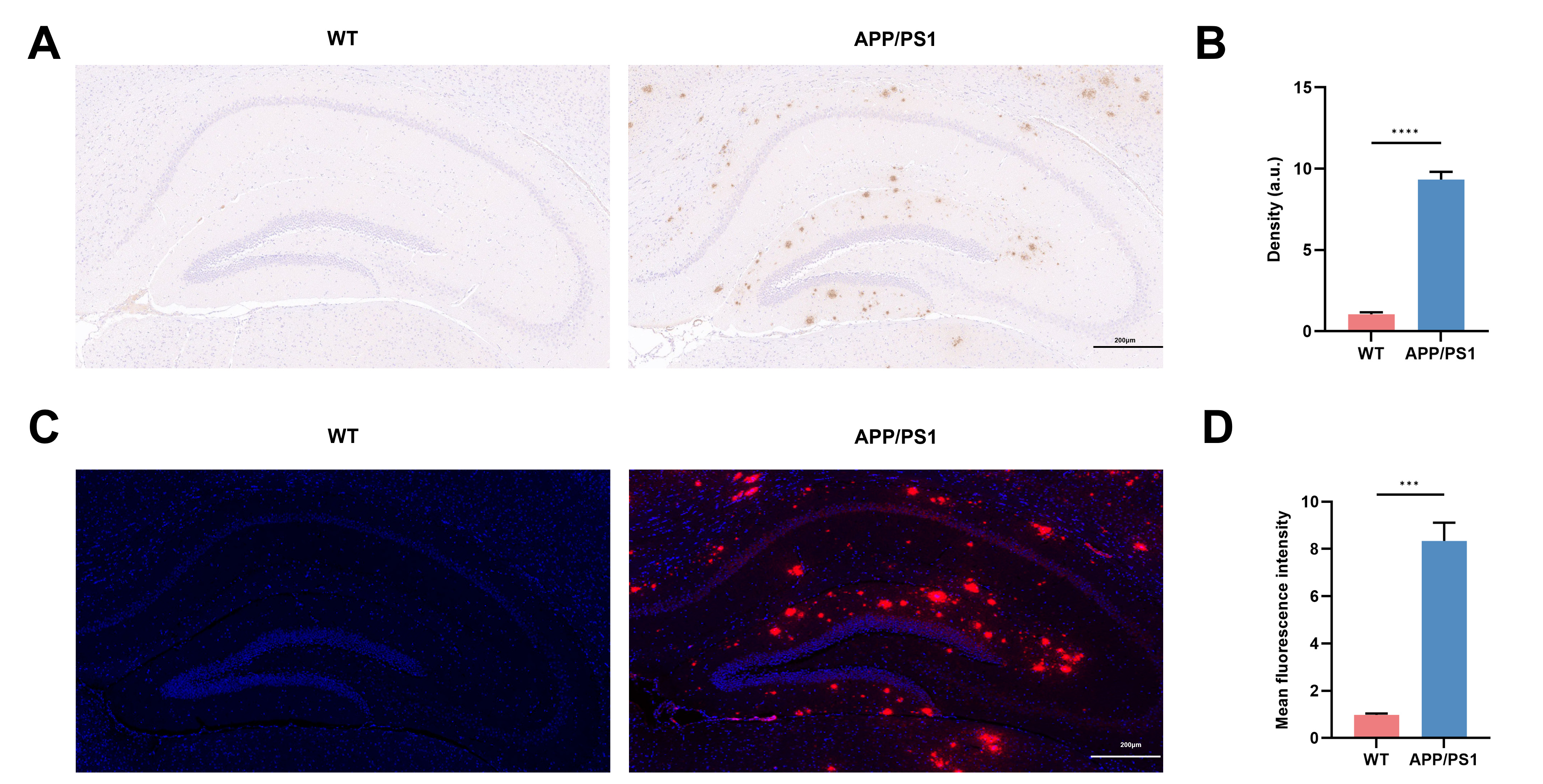


Supplementary Figure1. Immunohistochemical (IHC) and immunofluorescence (IF) analysis of Aβ deposition in the hippocampal region of mice from each group.

(A) IHC staining of Aβ in mice hippocampus. Scale bar = 200μm. (B) Quantification of the Aβ intensity area in (A) using ImageJ software. (n = 6). (C) Representative immunofluorescence images of Aβ in the hippocampal region. Scale bar = 200μm. (D) Quantification of the Aβ fluorescence intensity in (C) using ImageJ software. (n = 6). Data are presented as the mean ± SEM. ****P* < 0.001 and *****P* < 0.0001 vs. WT group.

## **Supplementary Figure2**


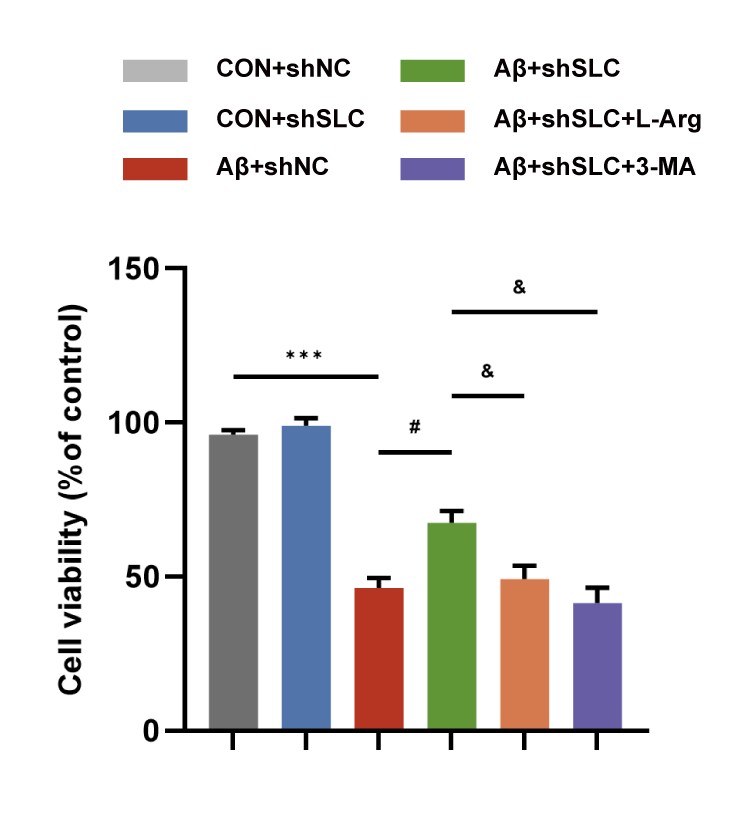


Supplementary Figure2. The viability of HT22 cells was determined by MTS assay.

The viability of HT22 cells was measured using an MTS assay. (n = 3). Data are presented as the mean ± SEM. ****P* < 0.001 vs. WT group; ^#^*P* < 0.05 vs the Aβ+shNC cells group; ^&^*P* < 0.05 vs. the Aβ+shSLC cells group.

## **Supplementary Figure3**


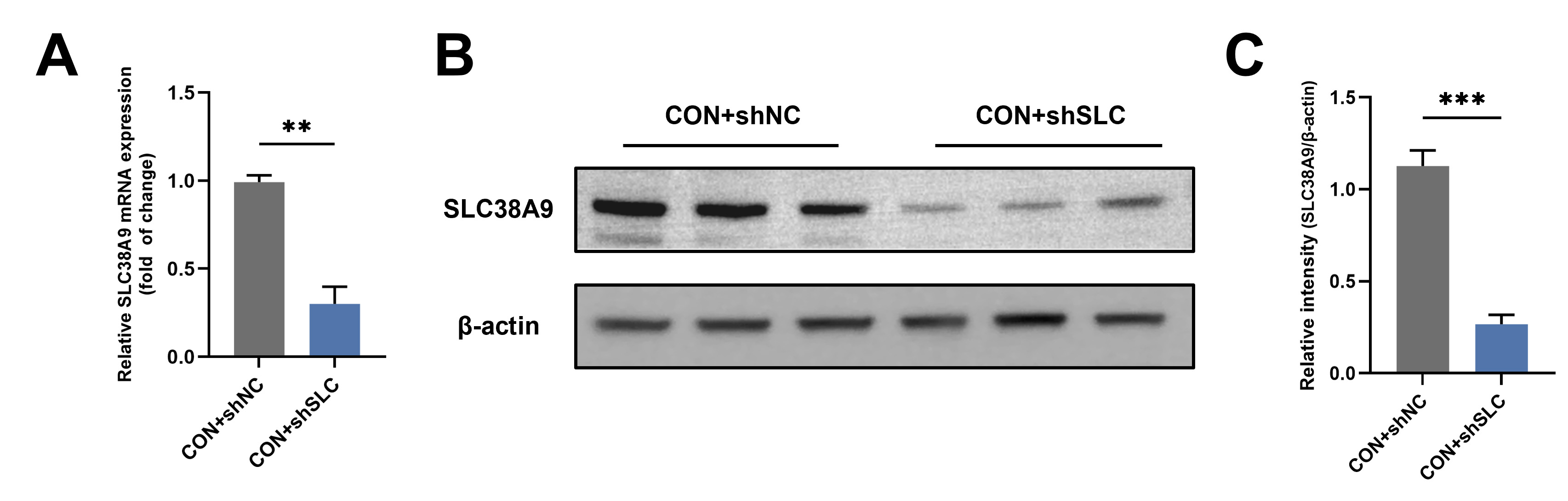


Supplementary Figure3. Detection of SLC38A9 expression levels in knockdown cells.

(A)The mRNA expression of SLC38A9 in knockdown cells were detected by RT-PCR. (n = 3). (B)-(C) Western blot analysis of SLC38A9 and β-actin in knockdown cells. Statistical analysis of Western blotting results, with β-actin as an internal reference. (n = 3). Data are presented as the mean ± SEM. ***P* < 0.01 and ****P* < 0.001 vs. CON+shNC group.

## **Supplementary Figure4**


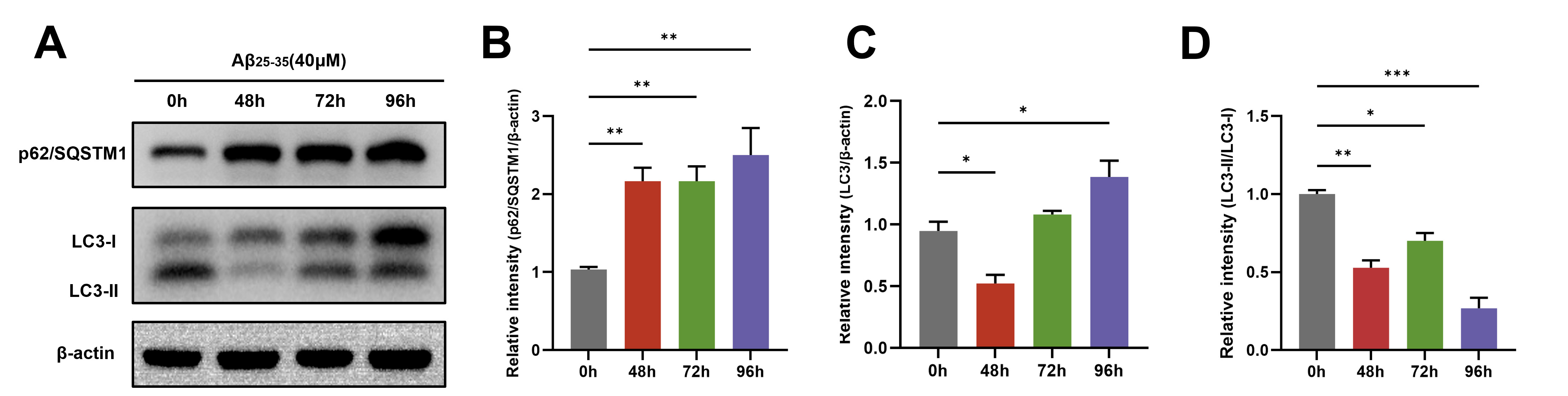


Supplementary Figure4. Temporal changes in autophagy markers following Aβ treatment.

(A) Western blot images of p62/SQSTM1, LC3 and β-actin levels in cells. (B-C) Statistical analysis p62/SQSTM1 and LC3 with β-actin as an internal reference of Western blotting results. (D) Statistical analysis LC3-II/I of Western blotting results. (n = 3). Data are presented as the mean ± SEM. **P* < 0.05, ***P* < 0.01 and ****P* < 0.001 vs. 0h group.

## **Supplementary Figure5**


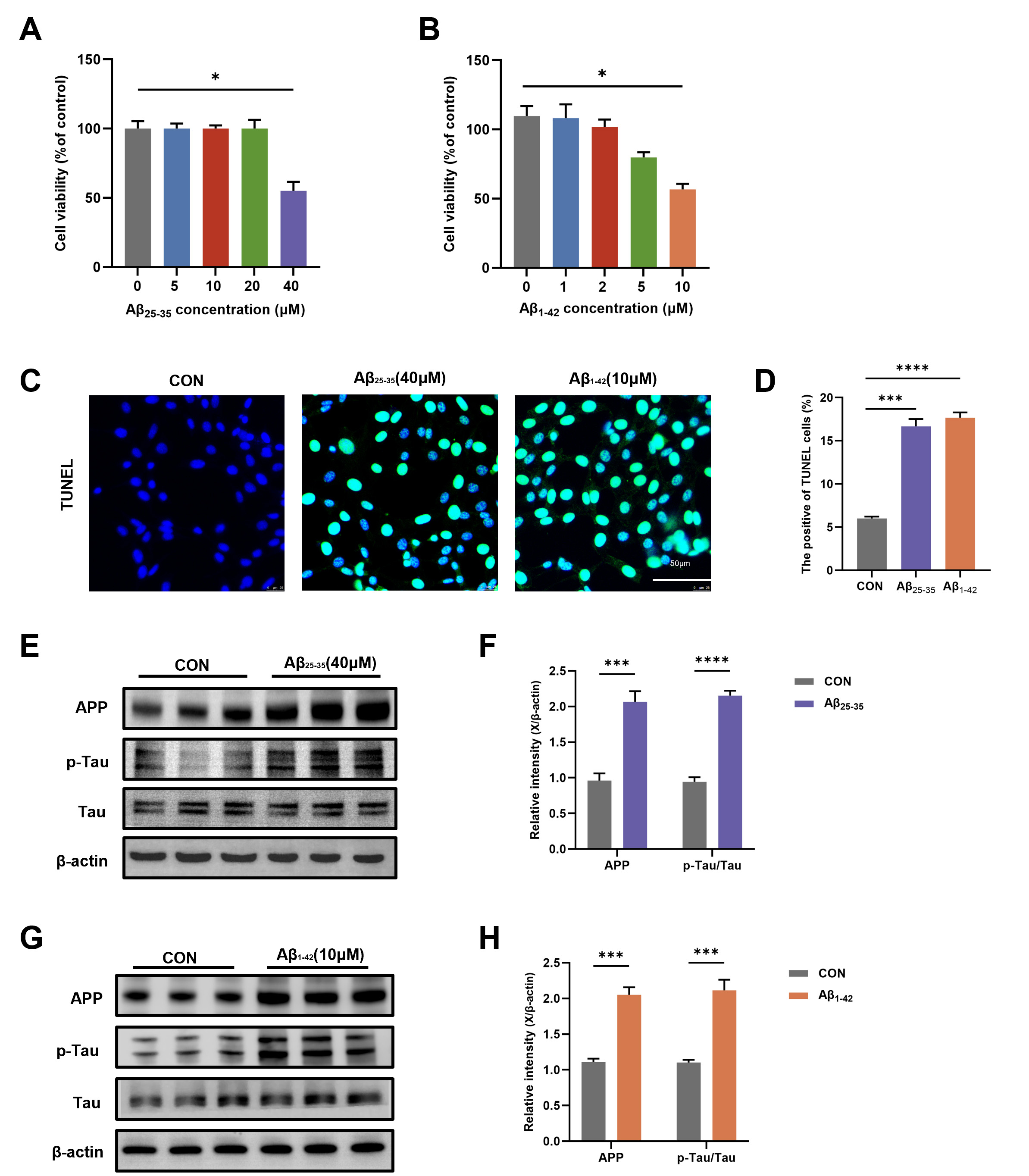


Supplementary Figure5. Evaluation of the cytotoxic effects of Aβ_1-42_ and Aβ_25-35_ and their impact on Alzheimer’s disease-related protein deposition in HT22 cells.

(A) The viability of HT22 cells was measured using a CCK-8 assay to explore the cytotoxicity of Aβ_25-35_ toward HT22 cells. Cells were treated with Aβ_25-35_ (0, 5, 10, 20, or 40 μM) for 48h. (B) The viability of HT22 cells was measured using a CCK-8 assay to explore the cytotoxicity of Aβ_1-42_ toward HT22 cells. Cells were treated with Aβ_1-42_ (0, 1, 2, 5, or 10 μM) for 48h. (C)-(D) Cell death was detected using a TUNEL (Green) assay. Quantification of the fluorescence intensity of HT22 cells using ImageJ software. (E)-(H) Western blot analysis of APP, p-Tau/Tau and β-actin. Statistical analysis of Western blotting results, with β-actin as an internal reference. (n = 3). Data are presented as the mean ± SEM. **P* < 0.05, ****P* < 0.001 and *****P* < 0.0001 vs. 0 μM group or CON group.

## **Supplementary Figure6**


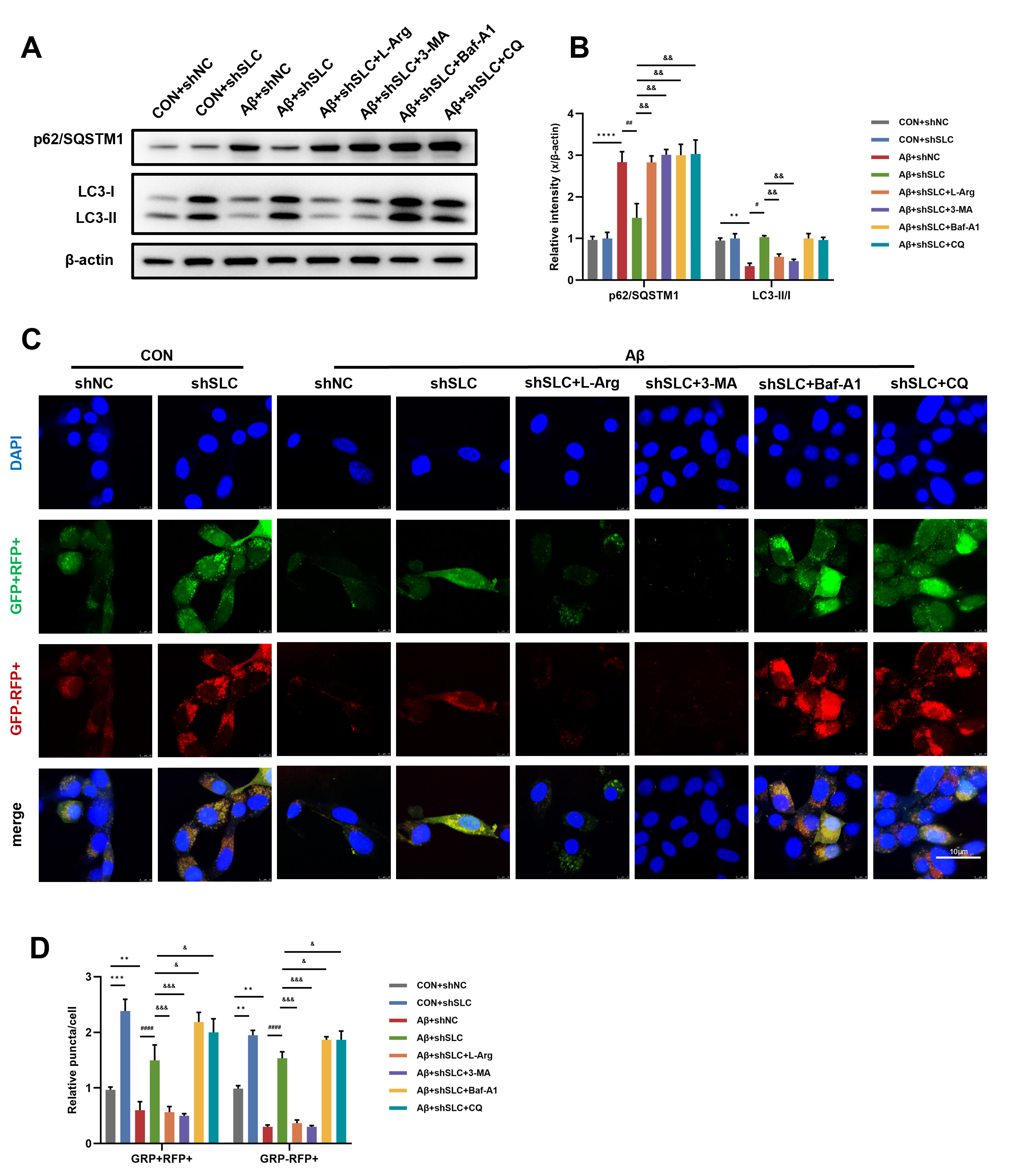


Supplementary Figure6. Validation of autophagic flux changes using bafilomycin A1 and chloroquine in the Aβ-treated cell model.

(A) Western blot analysis of p62/SQSTM1/β-actin and LC3-II/I. (B) Statistical analysis of Western blotting results. (n = 3). (C) Representative confocal images of LC3 puncta in HT22 cells expressing mRFP-GFP-LC3, scale bar = 10μm. (D) Quantification of the relative LC3 puncta in (C) using ImageJ software. (n = 3). Data are presented as the mean ± SEM. ***P* < 0.01, ****P* < 0.001, *****P* < 0.0001 vs. CON+shNC group; ^#^*P* < 0.05, ^##^*P* < 0.01, ^####^*P* < 0.0001 vs. the Aβ+shNC cells group; ^&^*P* < 0.05, ^&&^*P* < 0.01, ^&&&^*P* < 0.001 vs. the Aβ+shSLC cells group.

## **Supplementary Figure7**


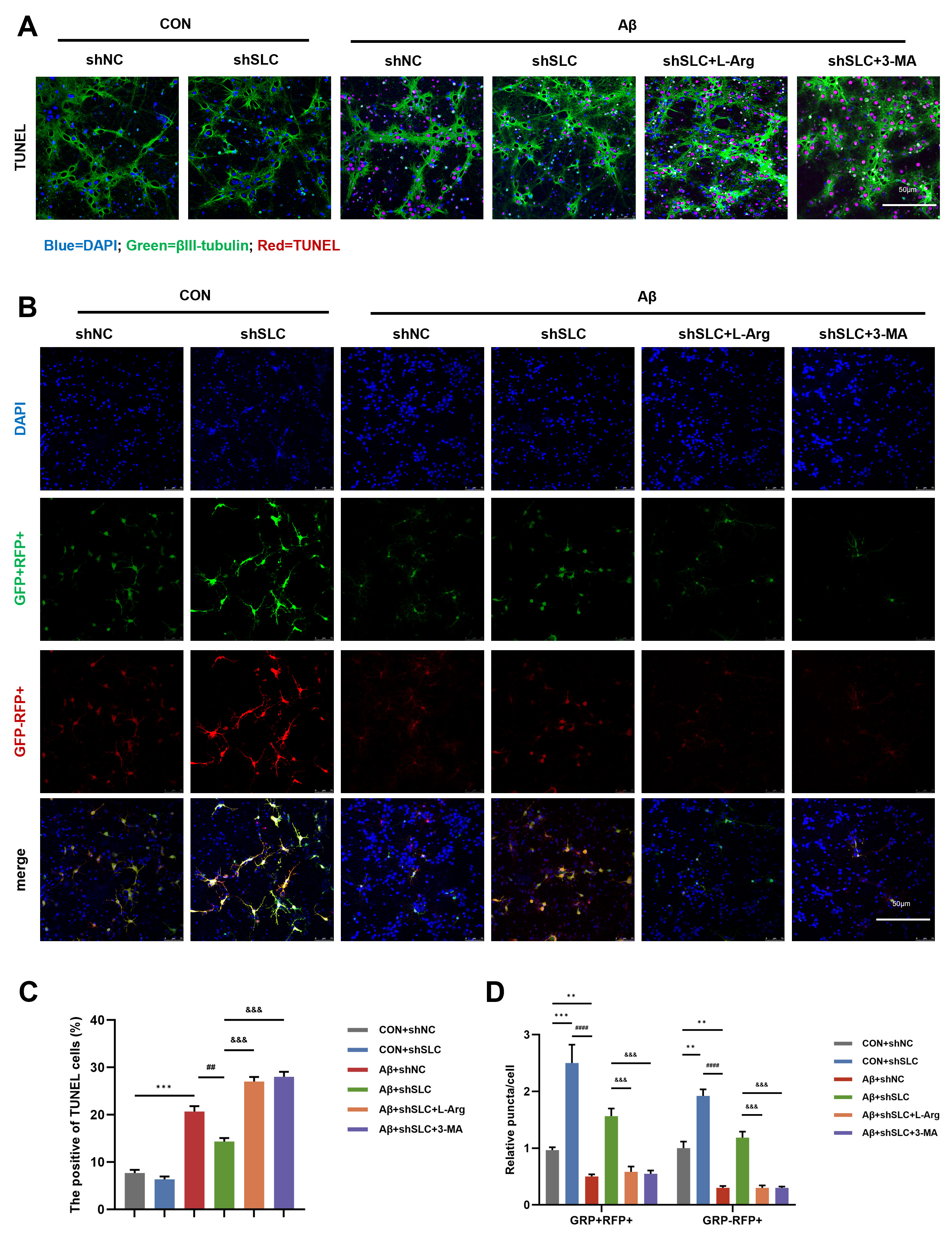


Supplementary Figure7. Validation of autophagy flux and apoptosis assays in mouse primary hippocampal neurons.

(A) Representative merged immunofluorescence images of βIII-Tubulin, TUNEL and DAPI in mouse primary hippocampal neurons cells. Scale bar = 50μm. (B) Representative confocal images of LC3 puncta in mouse primary hippocampal neurons cells expressing mRFP-GFP-LC3, scale bar = 50μm. (C) Quantification the proportion of TUNEL positive cells in (A) using ImageJ software. (n = 3). (D) Quantification of the relative LC3 puncta in (B) using ImageJ software. (n = 3). Data are presented as the mean ± SEM. ***P* < 0.01, ****P* < 0.001 vs. CON+shNC group; ^##^*P* < 0.01, ^####^*P* < 0.0001 vs. the Aβ+shNC cells group; ^&&&^*P* < 0.001 vs. the Aβ+shSLC cells group.

## **Supplementary Figure8**


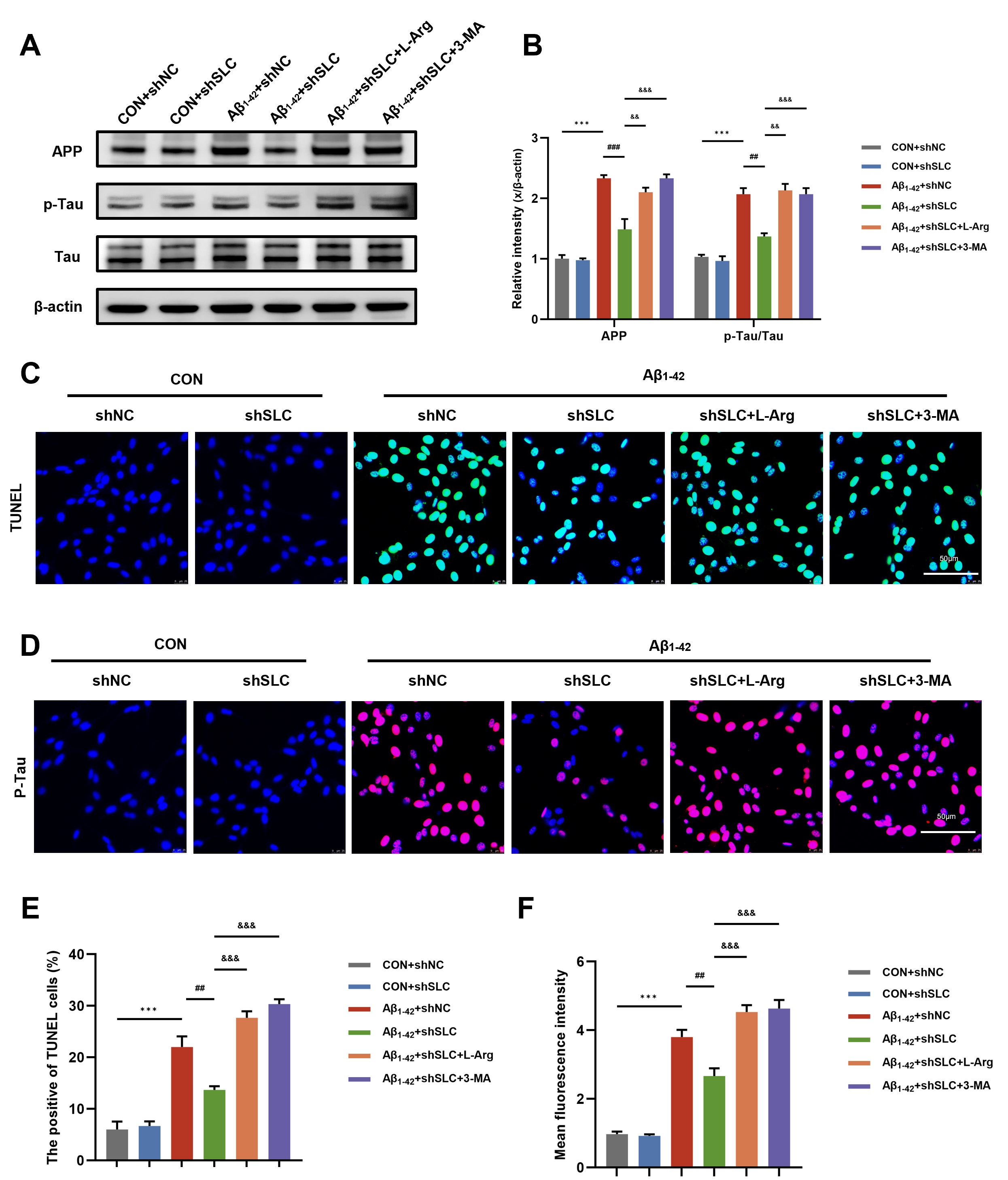


Supplementary Figure8. Effects of SLC38A9 knockdown on AD-related protein expression and cell apoptosis in Aβ_1-42_-treated HT22 cells.

(A) Representative immunoblotting bands of APP/β-actin and p-Tau/Tau in HT22 cells. (B) Statistical analysis of Western blotting results. (n = 3). (C) Representative merged immunofluorescence images of apoptosis cells (Green) and DAPI in HT22 cells. Scale bars = 50μm. (D) Representative merged immunofluorescence images of p-Tau (Red) and DAPI in HT22 cells. Scale bar = 50μm. (E) Quantification the proportion of TUNEL positive cells in (C) using ImageJ software. (n = 3). (F) Quantification of the fluorescence intensity in (D) using ImageJ software. (n = 3). Data are presented as the mean ± SEM. ****P* < 0.001 vs. CON+shNC group; ^##^*P* < 0.01, ^###^*P* < 0.001 vs the Aβ+shNC cells group; ^&&^*P* < 0.01, ^&&&^*P* < 0.001 vs. the Aβ+shSLC cells group.

## **Supplementary Figure9**


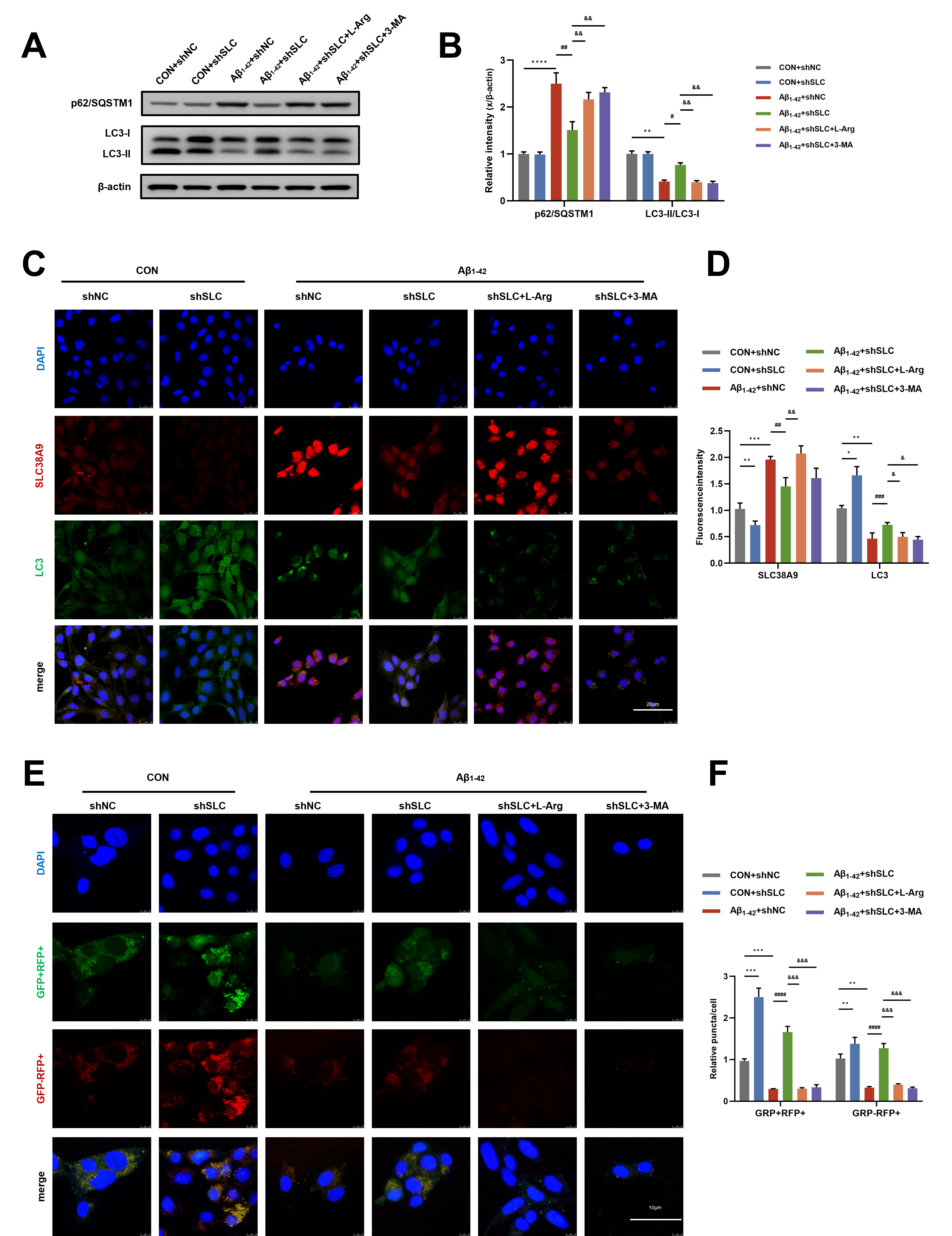


Supplementary Figure9. Effects of SLC38A9 knockdown on autophagic activity in Aβ_1-42_‑treated HT22 cells.

(A) Representative immunoblotting bands of p62/SQSTM1/β-actin and LC3-II/I in HT22 cells. (B) Statistical analysis of Western blotting results. (n = 3). (C) Representative merged immunofluorescence images of SLC38A9, LC3 and DAPI in HT22 cells. Scale bar = 20μm. (D) Quantification of the SLC38A9 and LC3 fluorescence intensity using ImageJ software. (n = 3). (E) Representative confocal images of LC3 puncta in HT22 cells expressing mRFP-GFP-LC3, scale bar = 10μm. (F) Quantification of the relative LC3 puncta in (E) using ImageJ software. (n = 3). Data are presented as the mean ± SEM. **P* < 0.05, ***P* < 0.01, ****P* < 0.001, *****P* < 0.0001 vs. CON+shNC group; ^#^*P* < 0.05, ^##^*P* < 0.01, ^###^*P* < 0.001, ^####^*P* < 0.001 vs the Aβ+shNC cells group; ^&^*P* < 0.05,^&&^*P* < 0.01, ^&&&^*P* < 0.001 vs. the Aβ+shSLC cells group.

## **Supplementary Figure10**


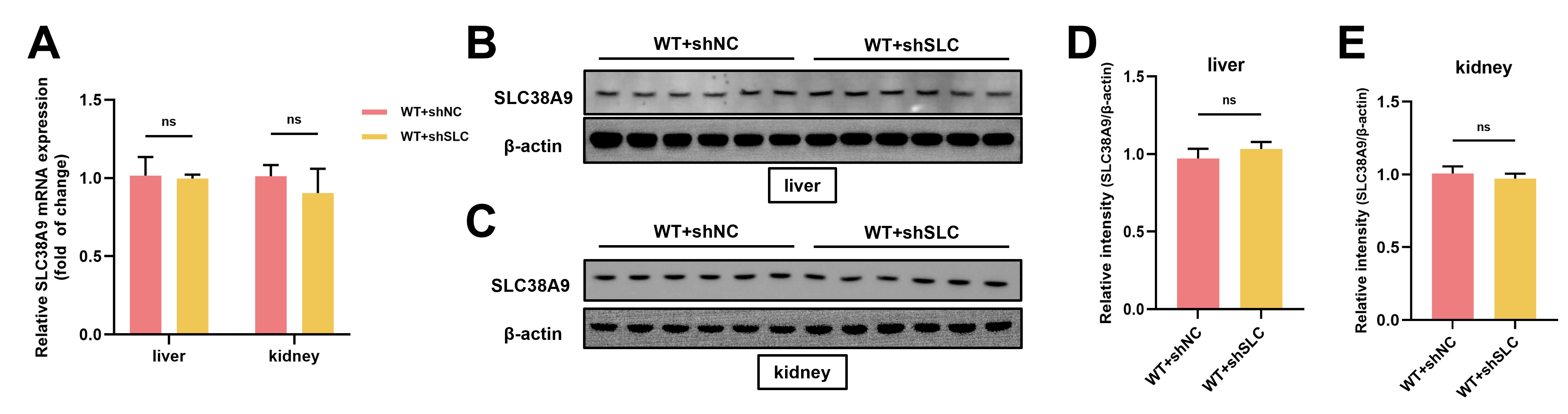


Supplementary Figure10. SLC38A9 expression in mice liver and kidneys after AAV‑BBB2.0 injection.

(A)The mRNA expression of SLC38A9 in WT+shNC group and WT+shSLC group were detected by RT-PCR. (n = 6). (B)-(E) Western blot analysis of SLC38A9 and β-actin in WT+shNC group and WT+shSLC group. Statistical analysis of Western blotting results, with β-actin as an internal reference. (n = 6). Data are presented as the mean ± SEM.

## **Supplementary Figure11**


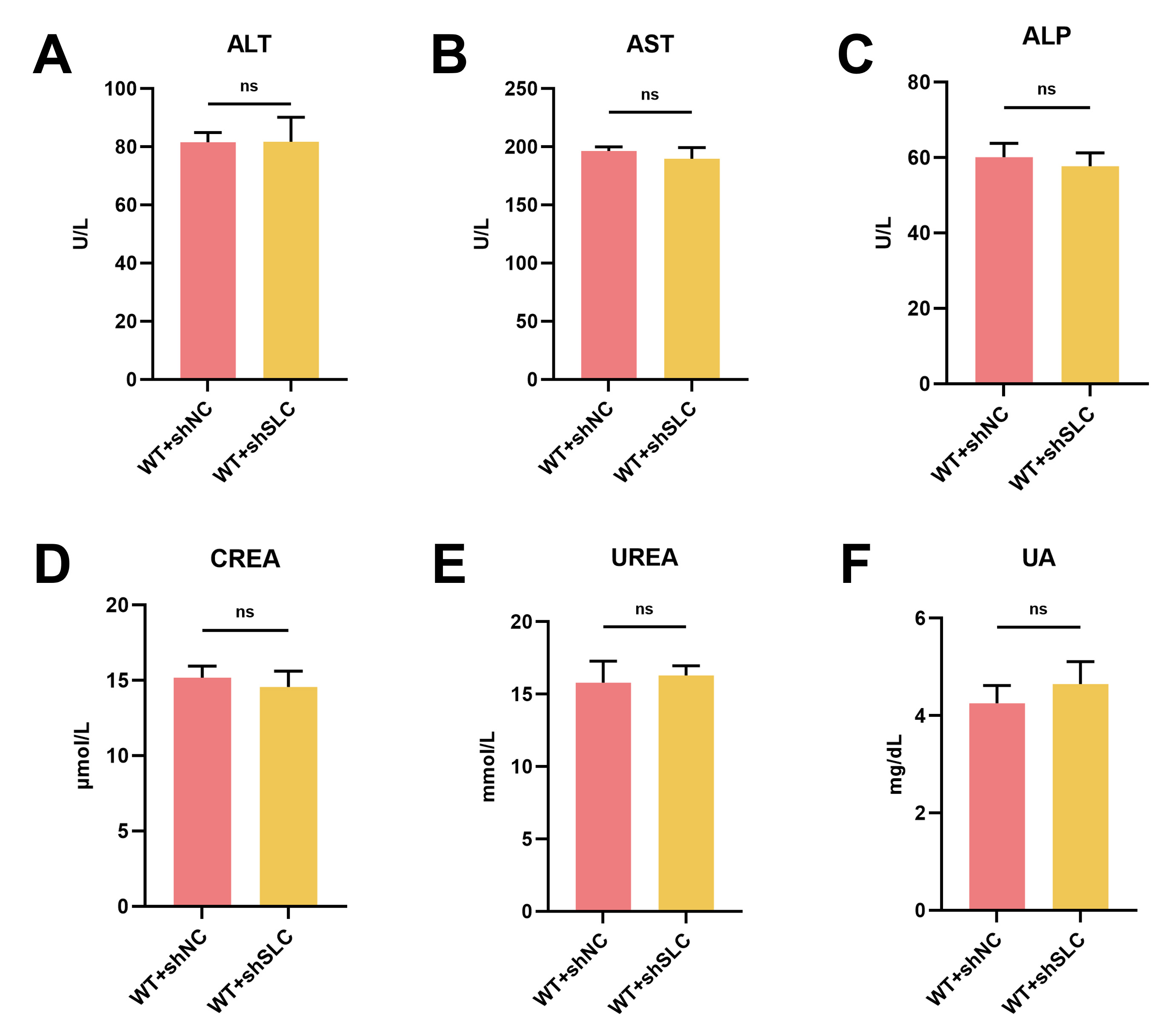


Supplementary Figure11. Serum biochemical indices of wild‑type mice treated with shNC and shSLC.

Serum biochemical indices of WT+shNC and WT+shSLC group mice. (A) ALT; (B) AST; (C) ALP; (D) CREA; (E) UREA; (F) UA. (n = 6). Data are presented as the mean ± SEM.

## **Supplementary Figure12**


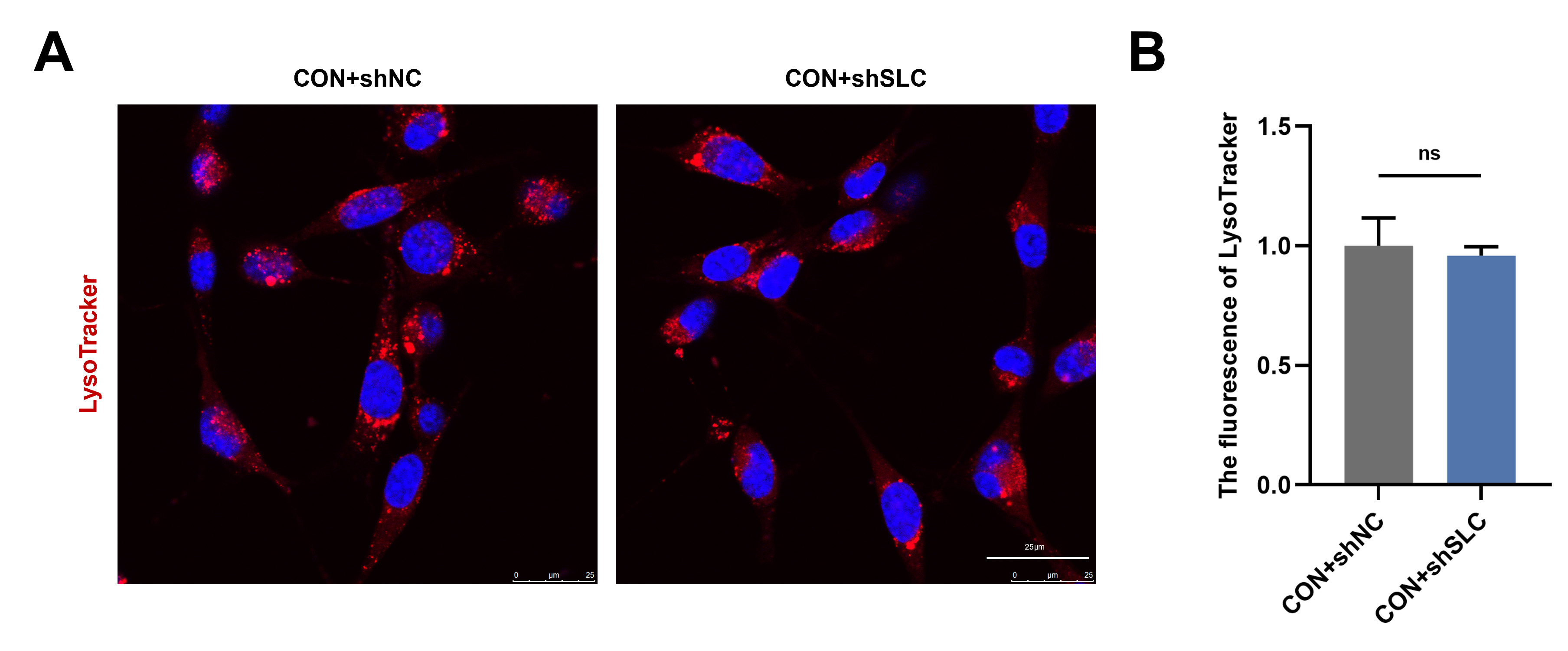


Supplementary Figure12. Assessment of lysosomal pH using LysoTracker staining.

(A) Lysosome pH values were assessed using LysoTracker Red. Scale bar = 25µm. (B) Quantification of the fluorescence intensity using ImageJ software. (n = 3). Data are presented as the mean ± SEM.

## **Supplementary Table1**. Antibodies

| **Antibody** | **Species** | **Vendor (City, State, catalogue)** |
| --- | --- | --- |
| APP | Rabbit | ABclonal (Wuhan, China) |
| p-Tau | Rabbit | ABclonal (Wuhan, China) |
| Tau | Rabbit | ABclonal (Wuhan, China) |
| PSD95 | Rabbit | Proteintech (Wuhan, China) |
| SYN | Rabbit | ABclonal (Wuhan, China) |
| P62 | Rabbit | ABclonal (Wuhan, China) |
| LC3 | Rabbit | Proteintech (Wuhan, China) |
| SLC38A9 | Rabbit | Proteintech (Wuhan, China) |
| p-mTOR | Rabbit | Proteintech (Wuhan, China) |
| mTOR | Rabbit | Proteintech (Wuhan, China) |
| p-ULK-1 | Rabbit | Proteintech (Wuhan, China) |
| ULK-1 | Rabbit | Proteintech (Wuhan, China) |
| p-p70S6K | Rabbit | Proteintech (Wuhan, China) |
| p70S6K | Rabbit | Proteintech (Wuhan, China) |
| β-actin | Rabbit | Servicebio (Wuhan, China) |
